# Viral Mimicry Response Is Associated With Clinical Outcome in Pleural Mesothelioma

**DOI:** 10.1016/j.jtocrr.2022.100430

**Published:** 2022-11-07

**Authors:** Suna Sun, Weihong Qi, Hubert Rehrauer, Manuel Ronner, Ananya Hariharan, Martin Wipplinger, Clément Meiller, Rolf Stahel, Martin Früh, Ferdinando Cerciello, Jean-François Fonteneau, Didier Jean, Emanuela Felley-Bosco

**Affiliations:** a Laboratory of Molecular Oncology, Department of Thoracic Surgery, University Hospital Zurich, Zurich, Switzerland; b Functional Genomics Center, ETH Zurich, University of Zurich, Zurich, Switzerland; c Centre de Recherche des Cordeliers, Inserm, Sorbonne Université, Université Paris Cité, Functional Genomics of Solid Tumors, Paris, France; d Coordinating Center, European Thoracic Oncology Platform (ETOP), Bern, Switzerland; e Swiss Group for Clinical Cancer Research (SAKK), Bern, Switzerland; f Department of Medical Oncology/Hematology, Cantonal Hospital of St. Gallen, St. Gallen, Switzerland; g Department of Medical Oncology, Inselspital, Bern University Hospital, University of Bern, Bern, Switzerland; h Nantes Université, Inserm UMR 1307, CNRS UMR 6075, Université d'Angers, CRCI2NA, Nantes, France

**Keywords:** Pleural mesothelioma, Endogenous retroviruses, Type I interferon, BRCA-associated protein 1

## Abstract

**Introduction:**

The aim of this study was to investigate endogenous retrovirus (ERV) expression and type I interferon (IFN) activation in human pleural mesothelioma (PM) and their association with clinical outcome.

**Methods:**

The expression of ERV was determined from PM cohorts and mesothelial precursor RNA sequencing data. The expression of ERV was confirmed by quantitative polymerase chain reaction (qPCR). Methylation of genomic DNA was assessed by quantitative methylation-specific PCR. DNA demethylation was induced in cells by demethylating agent 5-Aza-2’-deoxycytidine (5-Aza-CdR) treatment. To block type I IFN signaling, the cells were treated with ruxolitinib or MAVS silencing. The expression of IFN-stimulated genes (ISGs) was determined by qPCR and Western blot. Circulating ERVs were detected by qPCR.

**Results:**

Long terminal repeats (LTRs) represent the most abundant transposable elements up-regulated in PM. Within the LTR, *ERVmap_1248* and *LTR7Y*, which are specifically enriched in PM, were further analyzed. The 5-Aza-CdR treatment increased the levels of *ERVmap_1248* expression and induced *ERVmap_1248* promoter demethylation in mesothelial cells. In addition, *ERVmap_1248* promoter was more demethylated in the mesothelioma tissue compared with nontumor tissue. The 5-Aza-CdR treatment of the mesothelial cells also increased the levels of ISGs. Basal ISG expression was higher in the mesothelioma cells compared with the mesothelial cells, and it was significantly decreased by ruxolitinib treatment or MAVS silencing. Furthermore, ISG expression was higher in the tumor tissue with high expression levels of *ERVmap_1248*. High expression of *ERVmap_1248* was associated with longer overall survival and *BAP1* mutations. *ERVmap_1248* and *LTR7Y* can be detected in the PM plasma.

**Conclusions:**

We provide clues for patient stratification especially for immunotherapy where best clinical responses are associated with an activated basal immune response.

## Introduction

Pleural mesothelioma (PM) is a rapidly fatal disease arising from the monolayer tissue lining the walls of the pleural cavity and the internal organs housed inside.^1^ Major drivers include *CDKN2A/B*, the more PM-specific *BAP1*, and *NF2* mutations.^1^ Recent data suggest that subclonal *NF2* mutations may occur later in mesothelioma development.^2^^,^^3^ Traditionally, the major histologic types of mesothelioma have been the main histologic indicators of prognosis. Indeed, patients with sarcomatoid and biphasic tumors have substantial worse overall survival (OS) compared with patients with epithelioid tumors.^4^ Recent studies based on multiomics approaches^5^, ^6^, ^7^, ^8^ have refined the classification into four groups or into gradients based on molecular profiles.

PM is mostly associated with previous exposure to asbestos fibers,^1^ and we have recently found that exposure to asbestos in mice leads to increased expression levels of endogenous retrovirus (ERV) sequences.^9^

ERVs are integrated retroviral elements that cover 8% of the human genome.^10^ They are part of the so-called transposable elements (TEs), which include retrotransposons using RNA as an intermediate that is reverse transcribed into DNA and integrated in the genome and DNA transposons directly excising themselves from one location before reinsertion (reviewed in Wells et al.^11^). ERV and long-interspersed nuclear elements (LINEs) are autonomous retroelements encoding required proteins for retrotransposition, whereas short-interspersed nuclear elements (SINEs) and SINE-VNTR-Alu (SVA) elements require the machinery from autonomous retrotransposons.

Many ERV sequences are expressed during embryo development and are subsequently epigenetically silenced.^12^ Nevertheless, certain ERV sequences are actively transcribed and are elevated in cancer.^13^ Most ERVs in the human genome are nonautonomous long terminal repeat (LTR) elements that are either solitary (solo) LTR or LTR flanking a small segment of internal ERV sequences and are short in length. They are likely to serve as genomic regulators and affect the transcription in *cis*.^14^ The autonomous LTRs, however, consist of LTRs that flank potential protein-coding sequences and are near full-length proviral sequences, which could encode disease-associated antigens or functional RNA that regulated gene expression in *trans*.^14^ In addition to the effects as transcription regulators, the expression of ERV has been recently explored for its property as inducers of viral mimicry response, especially in immunotherapy context, and we and others have observed that expression of interferon-induced genes is associated with clinical outcome in patients with mesothelioma.^9^^,^^15^

Studying ERV expression has not been regularly implemented in high-throughput studies because repetitive elements are not frequently investigated and the analysis of the few RNA sequencing (RNA-seq) data in mesothelioma has mostly focused on the investigations of known genes.^5^^,^^7^^,^^16^ Cancer-specific LTR retroelements are mostly cancer type specific,^17^ and mesothelioma is one of the cancer types with the highest number of expressed cancer-specific LTR retroelement (eighth of 31 cancer types)^17^; however, for the time being, ERV expression in mesothelioma has not been thoroughly explored.

In this study, we extended previous work on ERV expression in human cancers^17^ and mesothelioma experimental animal models,^9^ and we reveal the expression of ERVs in human mesothelioma which can be detected in the blood and is associated with type I interferon (IFN) signaling and better OS.

## Materials and Methods

### ERV Analysis

Mesothelioma RNA-seq reads included in the analysis were the following: The Cancer Genome Atlas (TCGA) Mesothelioma Cohort (n = 87) downloaded from the National Center for Biotechnology Information database of genotypes and phenotypes in 2019, under phs000178.v10.p8; the Pleural Mesothelioma cohort from the Bueno study (n = 211) downloaded from the European Genome-phenome Archive (EGA) in 2020, under EGAS00001001563 (EGAD00001001915 and EGAD00001001916); the genetically characterized pleural mesothelioma primary cultures (FunGeST, n = 64) provided by Didier Jean’s team in 2022 for which RNA-Seq was performed as described in Meiller et al.^2^; and the human embryonic stem cell-derived mesothelium (n = 10) downloaded from the National Center for Biotechnology Information Gene Expression Omnibus in 2020, under GSE113090 (GSM3096389–GSM3096398). The choice of using human embryonic stem cell-derived mesothelium as control was dictated by the fact that RNA-Seq data on normal mesothelial cells or pleura are not available and ERV expression is cancer specific. RNA-seq reads were preprocessed using fastp (0.20.0). For analysis of transposable element (TE) expression, TE transcripts^18^ was used to obtain TE counts, followed by differential expression analysis using DESeq2. TE loci were considered to be significantly differentially expressed when the adjusted *p* values were less than 0.01, and where the log_2_ of the fold change was more than 1 for up-regulated loci and less than −1 for down-regulated loci. Full-length ERV sequences were downloaded from the ERVmap.^19^ The ERV expression was quantified using feature Counts in the Bioconductor package Rsubreads, where reads mapped uniquely to ERVs were counted. Mutational status of *BAP1*, *NF2*, and *CDKN2A* was extracted from the TCGA^7^ data set.

### PM Patients and Healthy Donors

Tumor tissue was collected from 155 patients with PM and nontumor tissue from six non-PM patients between 1999 and 2015^20^ (Supplementary Table 1A). The study was approved by the Ethical Committee Zürich (KEK-ZH-2012-0094 and BASEC-No. 2020-02566), and either patients signed informed consent or waiver of consent was granted by the Ethical Committee (BASEC-No. 2020-02566). The study methodologies were conformed to the standards set by the Declaration of Helsinki. Tissue samples were processed immediately for total RNA extraction or frozen as previously described.^20^ To assess that extracted RNA corresponded to tumor tissue containing at least 50% tumor content, we evaluated the PM score, which is based on *MSLN*, *CALB2*, and *PDPN* expression levels as previously described.^21^

Plasma was collected from 76 patients with PM between 2005 and 2012 and 42 healthy donors between 2002 and 2017 (Supplementary Table 1B). The patients with PM were enrolled in the trial SAKK 17/04 (NCT00334594).^22^ The study was approved by the Ethics Committee of the Zurich University Hospital (KEK-StV-Nr. 24/05), and either patients signed informed consent or waiver of consent was granted by the Ethical Committee (KEK-StV-Nr. 24/05). The study methodologies conformed to the standards set by the Declaration of Helsinki.

### Statistical Analysis

The figures represent the mean values from at least three independent experiments. Paired and unpaired *t* test, Mann-Whitney, chi-square test, Gehan-Breslow-Wilcoxon test, or Pearson correlation analysis was used and has been specified when used. Error bars indicate the SE of the mean. Statistical analysis was performed using Prism 8 (GraphPad 8.0.0).

### All Other Methods

Detailed description of the methods used is available in the Supplementary Methods.

## Results

### LTRs Represent the Most Abundant TEs Up-Regulated or Down-Regulated in PM Patient Data Sets

**Figure 1:**
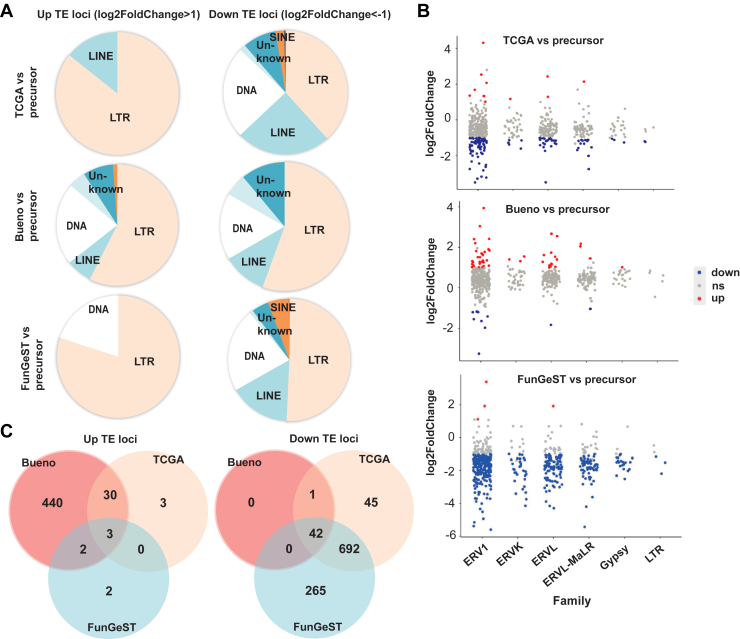
LTRs represent the most abundant TE up-regulated or down-regulated in data sets of patients with PM. (*A*) Pie charts revealing up- versus down-regulated TE loci. (*B*) TE loci up- or down-regulated in TCGA, Bueno, or FunGeST versus mesothelial precursor. Results were filtered by padj less than 0.01 and abs (log2Fold changes) greater than 1. Results are revealed for loci within the stated LTR families. (*C*) Overlap of the significantly up-regulated genes in the three comparisons visualized as Venn diagram. Significance was defined by padj less than 0.01. LTR, long terminal repeat; ns, expression not significantly changed; PM, pleural mesothelioma; TCGA, The Cancer Genome Atlas; TE, transposable element.

We used the RNA-seq data from TCGA,^7^ Bueno et al.’s^5^ tumor sample cohorts, or mesothelioma primary culture cell lines of FunGeST series^6^^,^^23^ and human embryonic stem cell-derived mesothelium^24^ as nontumor control, to determine which TE subfamilies are differentially expressed in PM tumors or primary cultures. We observed that LTRs represent the most abundant TEs up-regulated or down-regulated in PM patient data sets compared with mesothelial precursors. Furthermore, 86%, 57%, and 80% of the LTRs represent more than twofold up-regulated TEs and 38%, 56%, and 51% of the LTRs represent more than twofold down-regulated TEs in the TCGA, Bueno, and FunGeST data sets, respectively (Fig. 1*A* and Supplementary Table 3).

The ERV1 family has the highest number of up-regulated or down-regulated loci in LTR (Fig. 1*B*). Within the ERV1 family, *LTR48B*, *LTR7Y*, and *LTR6* were most often up-regulated in mesothelioma samples compared with mesothelial precursors (Fig. 1*C*, left panel). *LTR7Y* is an ERV specifically up-regulated in blastocyst stage of the human embryo.^25^ It is noteworthy that some *LTR7Y* up-regulated in blastocyst stage are located near genes such as *WNT16* and *FAM3C*, which are enriched in PM translatome^26^ or *NCF2* which is correlated to the so-called S-score, which defines the sarcomatoid component proportion of PM by transcriptome analysis^8^ (Supplementary Fig. 1). The ERV1 family constituted 43% of the most often down-regulated TE in mesothelioma samples compared with mesothelial precursors (Fig. 1*C*, right panel).

Next, we quantified locus-specific ERV expression comparing RNA-seq and reference ERV sequences using ERVmap,^19^ which uses stringent filtering criteria for RNA-seq reads that map to ERV loci and a 3220 ERV reference database of full-length proviral sequences.^27^

**Figure 2:**
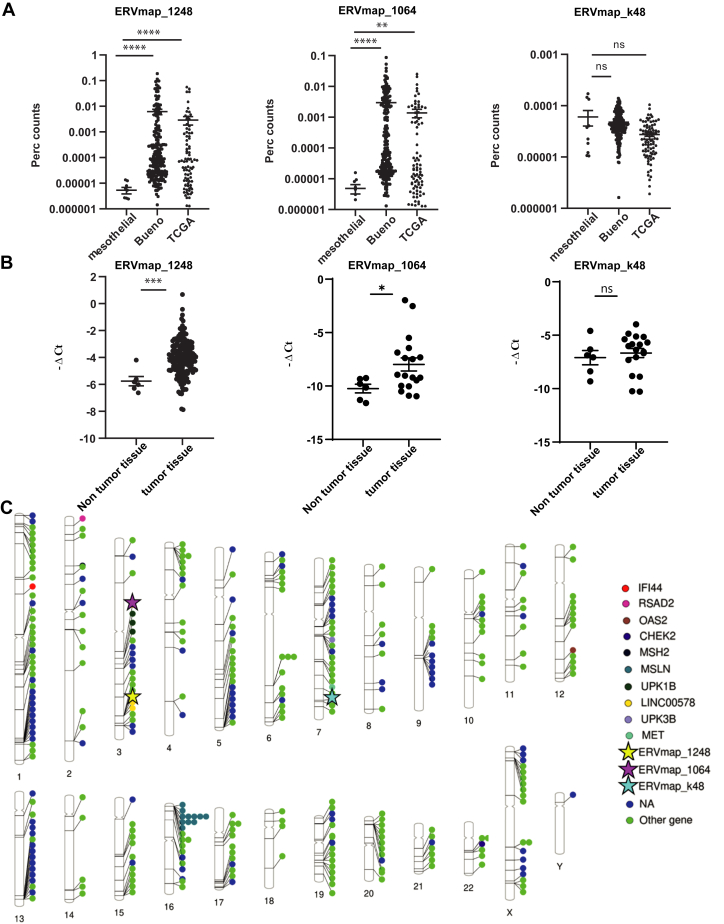
PM-specific ERV. (*A*) Expression of two representative ERV sequences *ERVmap_1248* and *ERVmap_1064* increases in tumors but not the control *ERVmap_k-48*. (∗) *p* less than 0.05; (∗∗) *p* less than 0.01; (∗∗∗) *p* less than 0.001; (∗∗∗∗) *p* less than 0.0001; ns, not significant, Mann-Whitney test. (*B*) RT-qPCR validation of ERV expression in samples from human nontumor tissue and tumor tissue. (∗) *p* less than 0.05; (∗∗) *p* less than 0.01; (∗∗∗) *p* less than 0.001; ns, not significant, Mann-Whitney test. (*C*) Location of ERVmaps and other ERVs with enriched expression in PM. The ideogram was created using http://visualization.ritchielab.org/phenograms/plot. ERV, endogenous retrovirus; PM, pleural mesothelioma; RT-qPCR, quantitative reverse-transcriptase polymerase chain reaction.

We were particularly interested to full-length proviral sequences because it has recently been suggested that viral mimicry primed cancers include elevated baseline expression of retrotransposons that form double-stranded RNA (dsRNA) and elevated levels of retrotransposon-derived antigenic peptide that forms tumor-associated antigens.^28^ We stratified the ERVmaps by their total counts and specific enrichment in tumor. Two representative ERVs—*ERVmap_1248* (hg38, chr3: 177,657,043–177,671,140) and *ERVmap_1064* (hg38, chr3: 112,413,019–112,423,381)—both belonging to ERVH group of the ERV1 family, were identified based on their enrichment in tumors. Another ERV sequence, *ERVmap_k48* (also called *HERV-K15*^29^), where counts almost do not differ between PM cohorts and precursors, was also selected as control (Fig. 2*A*). Coverage of *ERVmap_1248* locus was also enriched in FunGeST series, however to a lower extent (Supplementary Fig. 2*A*), likely due to the polyA RNA-seq protocol used which has been found to fail to detect several classes of repeat RNA.^30^ Of note, the patients expressing levels of *ERVmap_1248* above the average were enriched for *BAP1* mutations but not for *NF2* or CDKN2A mutations (Supplementary Fig. 2*B*). We confirmed that the expressions of *ERVmap_1248* and *ERVmap_1064* but not of the control *ERVmap_k48* were lower in normal compared with tumor tissue (Fig. 2*B*). The difference was maintained when females were excluded (Supplementary Fig. 2*C*). We next compared the chromosomal location of the two ERVmaps enriched in mesothelioma with the chromosomal location of ERVs specifically expressed in mesothelioma extracted from a pan-cancer analysis.^17^ Of note, 28 of 357 (8%) of those ERVs are located near genes relevant for mesothelioma, such as *MSLN*, *MET*, *UPKB1*, *UPK3B*, LINC00578, some interferon-stimulated genes (*RSAD2*, *IFI44*, *OAS2*), or DNA damage-related genes (*CHEK2*, *MSH2*) (Fig. 2*C*). *MSLN* locus on chromosome 16 is especially enriched in ERV expression in mesothelioma (11 ERVs). The ERV maps enriched in mesothelioma that we identified were located in ERV expression locations previously described^17^ (Fig. 2*C*).

### ERVmap_1248 Can Be Induced by 5-Aza-2’-Deoxycytidine Treatment in Mesothelial Cells

**Figure 3:**
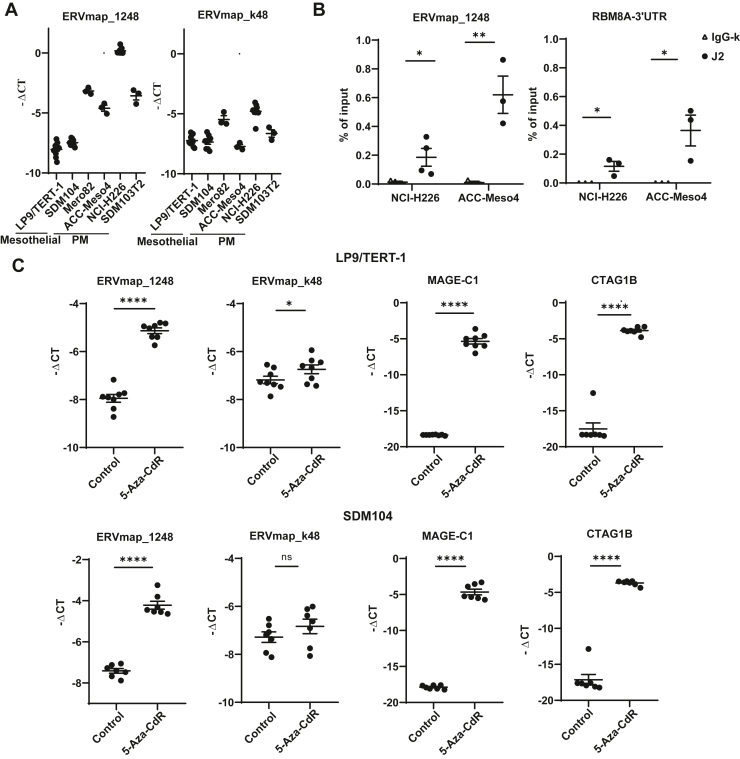
*ERVmap_1248* expression is lower in normal mesothelial cells compared with the PM cells, it can form dsRNA and its expression can be induced by 5-Aza-CdR in the mesothelial cells. (*A*) Basal expression of *ERVmap_1248* and *ERVmap_k48* in human PM cells and human mesothelial cells. (*B*) RT-qPCR analysis of *ERVmap_1248* and *RNA Binding Motif 8A* (*RBM8A*)-3’UTR transcripts captured by J2 antibody in pulldown assay, (∗) *p* less than 0.05, (∗∗) *p* less than 0.01, two-tailed paired *t* tests. RBM8A-3’UTR is used as a positive control. (*C*) ERVmap_1248 expression increased significantly with 5-Aza-CdR treatment in LP9/TERT-1 and SDM104 mesothelial cells, whereas ERVmap_k48 expression increased by approximately 50% in LP9/TERT1 but not in SDM104 cells. (∗) *p* less than 0.05; (∗∗∗∗) *p* less than 0.0001; ns, not significant, two-tailed paired *t* test. MAGE-C1 and CTAG1B are used as positive controls (36). 5-Aza-CdR, 5-Aza-2’-deoxycytidine; dsRNA, double-stranded RNA; PM, pleural mesothelioma; RT-qPCR, quantitative reverse-transcriptase polymerase chain reaction.

Among the selected ERVs, the most abundant one, *ERVmap_1248*, and the control ERV, *ERVmap_k48*, were further analyzed. Basal expression of *ERVmap_1248* and *ERVmap_k48* was investigated in human mesothelial cells LP9/TERT-1 and SDM104 and in human PM cells Mero82, ACC-Meso4, NCI-H226, and SDM103T2, used as surrogate for normal tissue versus mesothelioma, respectively. The expression of *ERVmap_1248* is on average 16-fold more enriched in PM cells compared with *ERVmap_k48*, supporting the concept of *ERVmap_1248* activation in mesothelioma (Fig. 3*A*). We had previously found that ERVs which are expressed in mouse mesothelioma cells form dsRNA^9^ and *ERVmap_1248* is also predicted to form dsRNA (Supplementary Fig. 3). This was confirmed (Fig. 3*B*) by RNA pull-down experiments using J2 anti-dsRNA antibody. *RNA Binding Motif 8A* (*RBM8A*)-3’UTR which we have previously found to be a substrate for dsRNA editing^31^ was used as a positive control (Fig. 3*B*).

Next, we aimed at investigating whether promoter demethylation was a possible cause for increased *ERVmap_1248* expression in PM as previously observed with murine mesothelioma ERV.^9^ Therefore, DNA demethylation was induced in human mesothelial cells LP9/TERT-1 and SDM104 by treatment with 5-Aza-2’-deoxycytidine (5-Aza-CdR), which is a DNA methyltransferase inhibitor. This treatment resulted in a significantly eightfold increase in *ERVmap_1248* expression whereas the control ERV *ERVmap_k48* increased by approximately 50% in LP9/TERT1 but not in SDM104 cells (Fig. 3*C*). The expression of two cancer-associated testis antigens *CTAG1B* and *MAGE-C1* genes was used as positive controls as we previously described.^32^

### Basal Levels of ISG Expression Are Higher in PM Cells With Intact IFNB1 Coding Gene and 5-Aza-CdR Increases ISG Expression in Mesothelial Cells and It Is Associated With ERVmap_1248 Promoter Demethylation

**Figure 4:**
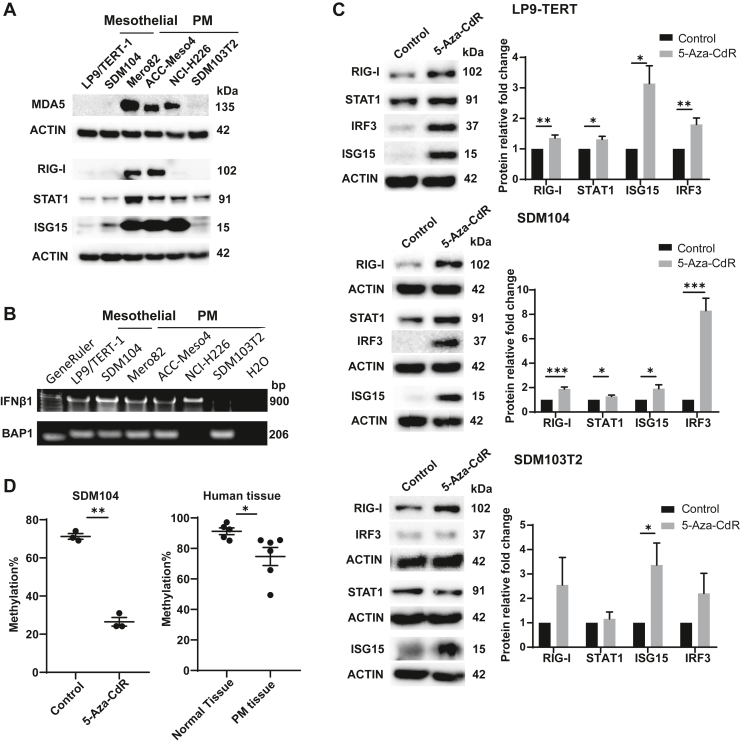
Basal ISG expression is higher in the PM cells with intact IFN coding genes and 5-Aza-CdR increased ISGs in mesothelial cells and is associated with promoter demethylation. (*A*) The expression of ISGs MDA5, RIG-I, STAT1, and ISG15 increased in the PM cells except SDM103T2 cells on protein level. (*B*) PCR-mediated detection of IFNB1 and BAP1 genes using genomic DNA from mesothelial and PM cells. The PCR fragment of BAP1 was used as DNA input control. (*C*) The expression of RIG-I, STAT1, IRF3, and ISG15 all increased on 5-Aza-CdR treatment in LP9/TERT-1 and SDM104 mesothelial cells but not all in SDM103T2 PM cells. WB quantification: n = 4–12. (∗) *p* less than 0.05; (∗∗) *p* less than 0.01; (∗∗∗) *p* less than 0.001, two-tailed paired *t* test. (*D*) The fraction of ERVmap_1248 promoter methylation decreased after 5-Aza-CdR treatment of SDM104 cells (left panel). The human PM tissue has less methylation percentage in ERVmap_1248 promoter compared with normal tissue. (∗) *p* less than 0.05; (∗∗) *p* less than 0.01, Mann-Whitney test. 5-Aza-CdR, 5-Aza-2’-deoxycytidine; IFN, interferon; PCR, polymerase chain reaction; PM, pleural mesothelioma.

To assess whether differential *ERVmap_1248* expression between mesothelial and PM cells is associated with a differential type I IFN signaling activation as observed in the mouse model,^9^ we investigated the basal expression levels of various interferon-stimulated genes (ISGs).^33^ We observed that MDA5, RIG-I, STAT1, and ISG15 levels are higher in most PM cells when compared with mesothelial cells (Fig. 4*A*). Low levels of RIG-I in H226 cells are likely due to the mutation in RIG-I–encoding *DDX58* gene resulting in G191_K193 duplication at the end of the second caspase activation and recruitment domain (http://www.cbioportal.org/). In addition, in PM cell line SDM103T2, ISG levels were at the same level as mesothelial cells (Fig. 4*A*). We have previously found that basal type I IFN signaling is present in cells with intact *IFNB1* gene in PM.^34^ Accordingly, deficiency of *IFNB1* gene was observed in SDM103T2 but not the other cell lines (Fig. 4*B*). A polymerase chain reaction (PCR) fragment covering exon 5 of *BAP1* was used as control for genomic DNA input during PCR (Fig. 4*B*). BAP1 is known to be deleted in the NCI-H226 cells.^35^

We next assessed whether demethylation, which results in increased *ERVmap_1248* expression in mesothelial cells, is also associated with increased ISG levels. As expected, we observed increased levels of RIG-I, STAT1, IRF3, and ISG15 in mesothelial cells LP9/TERT-1 and SDM104 on 5-Aza-CdR treatment. *IFNB1*-deficient PM cell line SDM103T2 was used as control. In this cell line, the increase of gene expression of *CTAG1B* and *MAGE-C1* was observed as expected (Supplementary Fig. 4); however, we observed no significant increase of IFN-dependent STAT1, RIG-I, or IRF3. Only the expression of IFN-independent^36^ ISG15 was significantly increased (Fig. 4*C*).

To confirm that the increase in *ERVmap_1248* expression observed upon treatment with 5-Aza-CdR is due to promoter demethylation, we first identified the target region based on the analysis of CpG islands (Supplementary Fig. 5) to design methylation-specific primer “M” and unmethylation-specific primer “U” for the region of the promoter of *ERVmap_1248*. These primers were used on sodium bisulfite-treated DNA, where all methyl-free cytosines are converted into uracils, whereas methylated cytosines remain unchanged allowing the use of quantitative methylation-specific PCR.

In the SDM104 cells, *ERVmap_1248* promoter methylation decreased significantly upon 5-Aza-CdR treatment, and we also observed that the *ERVmap_1248* promoter is significantly more demethylated in the tumor tissues compared with the nontumor tissues (Fig. 4*D*).

### Expression of ISG Can Be Decreased in Mesothelioma Cells by Treatment With JAK Inhibitor Ruxolitinib and MAVS Silencing

**Figure 5:**
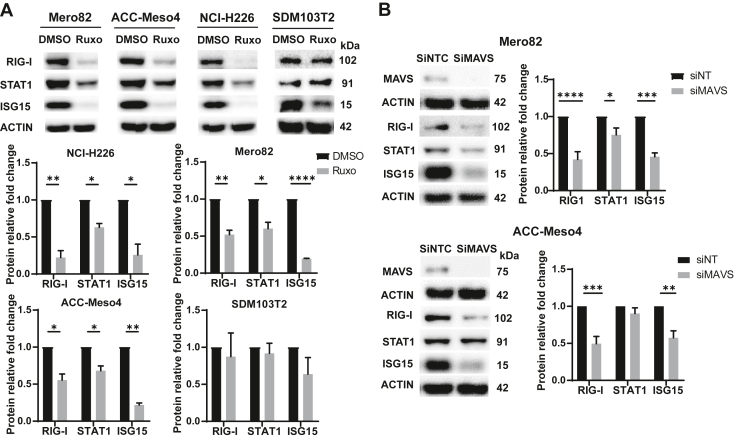
Basal levels of ISG can be decreased by treatment with JAK inhibitor ruxolitinib and MAVS silencing. (*A*) The expression of ISGs RIG-I, STAT1, and ISG15 decreased after ruxolitinib (Ruxo), a JAK1/2 inhibitor treatment in the PM cells except SDM103T2 cells. WB quantification: n = 3–4. (∗) *p* less than 0.05; (∗∗) *p* less than 0.01; (∗∗∗∗) *p* less than 0.0001, two-tailed paired *t* test. (*B*) Silencing MAVS decreased ISGs RIG-I, STAT1, and ISG15 levels in Mero82 and ACC-Meso4 PM cells. WB quantification: n = 3. (∗) *p* less than 0.05; (∗∗) *p* less than 0.01; (∗∗∗) *p* less than 0.001; (∗∗∗∗) *p* less than 0.0001, two-tailed paired *t* test. PM, pleural mesothelioma.

To verify the activation of type I IFN signaling, PM cells were treated with ruxolitinib, a JAK1/2 inhibitor blocking the type I IFN signaling. This treatment resulted in decreased levels of ISG in the PM cell lines NCI-H226, Mero82, and ACC-Meso4 with an intact *IFNB1* gene but not in *IFNB1* gene-deficient cell line SDM103T2 (Fig. 5*A* and Supplementary Fig. 6). To confirm the involvement of dsRNA sensing, we silenced MAVS, which is downstream of the activation of dsRNA sensors RIG-I and MDA5. Silencing MAVS in the PM cells Mero82 and ACC-Meso4 resulted in a significant decrease of RIG-I, STAT1, and ISG15 (Fig. 5*B*).

Altogether, these data indicate that increased ISG expression is dependent on type I IFN receptor signaling.

### ISG Expression Is Higher in ERVmap_1248 High PM Tissues and Patients With Higher ERVmap1248 Expression Have Better Survival

**Figure 6:**
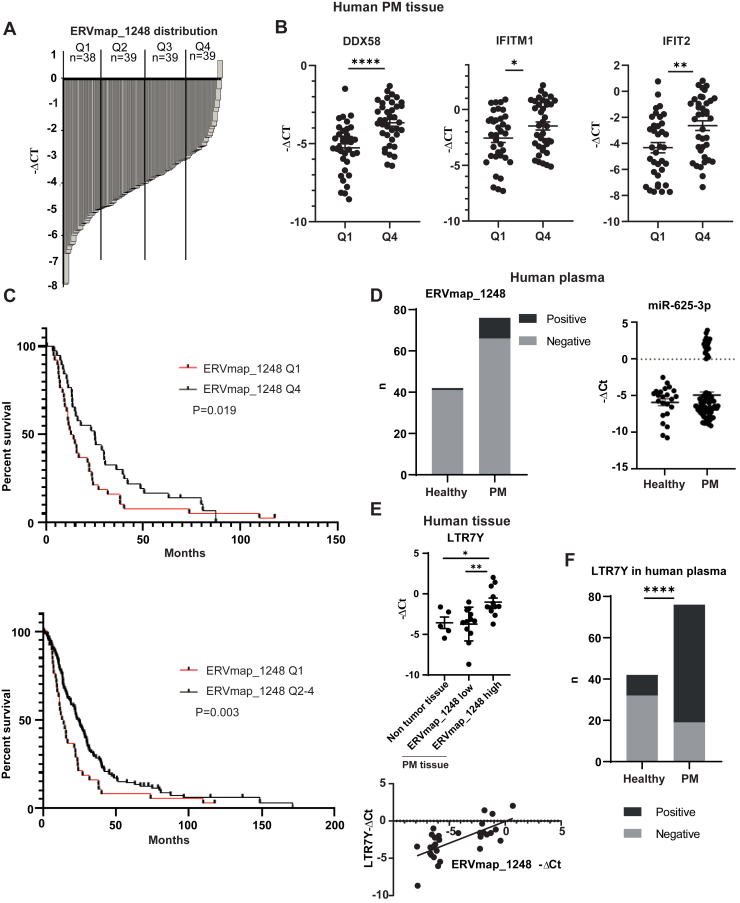
*ERVmap_1248* high expression is associated with clinical outcome in PM. (*A*) *ERVmap_1248* expression distribution in tumor tissues from patients with PM. (*B*) The expression of ISGs *DDX58*, *IFITM1*, and *IFIT2* is higher in *ERVmap_1248* high tumor tissues. (∗) *p* less than 0.05; (∗∗) *p* less than 0.01; (∗∗∗∗) *p* less than 0.0001, two-tailed unpaired *t* test. (*C*) Kaplan-Meier curves of overall survival according to *ERVmap_1248* expression in patients with PM. Red and black curves represent lower (Q1) or higher *ERVmap_1248* (Q4 or Q2–4) expression, respectively. Overall survival was calculated from date of diagnosis. Patients with PM from Q2 or Q2 to 4 have better survival rate than those from Q1. Gehan-Breslow-Wilcoxon test. (*D*) *ERVmap_1248* detection in the plasma of patients with PM and healthy volunteers. Detection of *miR-625-3p* is used as comparison. (*E*) *LTR7Y* and *ERVmap_1248* expressions are correlated. (∗) *p* less than 0.05; (∗∗) *p* less than 0.01, two-tailed unpaired *t* test. Correlation: R = 0.6798, *p* less than 0.0001, Pearson correlation analysis. (*F*) *LTR7Y* detection in the plasma of patients with PM and healthy volunteers. (∗∗∗∗) *p* less than 0.0001, chi-square test. PM, pleural mesothelioma; Q, quarter.

Patients with PM were distributed into four groups (Q1–Q4) based on their *ERVmap_1248* expression (Fig. 6*A*). Q1 has lowest and Q4 has highest *ERVmap_1248* expression. The expression of ISGs, which we previously have associated with better OS,^9^ was higher in the tumor tissues with high expression levels of *ERVmap_1248* (Fig. 6*B*). Furthermore, *ERVmap_1248* expression was analyzed in relation to survival. OS was longer among patients with higher *ERVmap_1248* expression (Q4 or Q2–4) compared with patients whose tumors had lower *ERVmap_1248* expression (Q1) (Q4 versus Q1: median OS 25.16 versus 13.55 mo, hazard ratio = 1.57, *p* = 0.019; Q2–4 versus Q1: median OS 24.51 versus 13.55 mo, hazard ratio = 1.761, *p* = 0.003) (Fig. 6*C*). This observation was confirmed using the TCGA and Bueno data sets (Supplementary Fig. 7A) and is consistent with the TCGA cluster classification where the cluster with the best survival is related with type I IFN signaling and *BAP1* mutation is associated with a longer overall patient survival rate.^15^ No significant association with histotype was observed (Supplementary Fig. 7B). To increase the clinical relevance of *ERV-map1248* expression, we next assessed how its expression was associated with the four-gene (*CD8A*, *STAT1*, *LAG3*, and *CD274*) inflammatory signature score, which has recently been described to predict response to checkpoint inhibitors in the CheckMate743 trial.^37^ The four-gene signature score was significantly higher in Q4 compared with the Q1 group (Supplementary Fig. 7C).

Finally, using plasma collected in the SAKK 17/04 (NCT00334594)^22^ trial, we determined that circulating *ERVmap_1248* (Fig. 6*D*) can be detected in a small fraction (13%) of patients with PM, whereas only one normal control was positive of 42 normal plasma (2%). Relative levels could not be assessed due to difference in levels of the two normalizers used (Supplementary Fig. 8A). Circulating *ERVmap_1248* was independent of the age of the patients (Supplementary Fig. 8B). The quality of RNA was assessed by quantification of relative levels of miR-625-3p where the levels of miR-16 used as normalizer were not different between healthy volunteers and patients with PM (Supplementary Fig. 8C). The bimodal detection of *miR-625-3p* previously described^38^ was observed only in the patients with PM (Fig. 6*D*).

We reasoned that the low sensitivity of ERV detection might be due to ERVmaps being full-length proviral sequences where the size is longer compared with solo-LTR. Therefore, we investigated whether *LTR7Y* expression could be used as circulating ERV instead of *ERVmap_1248*. We first determined that *LTR7Y* expression is enriched in the PM tissues with high *ERVmap_1248* expression and that *LTR7Y* levels are significantly correlated with *ERVmap1248* (Fig. 6*E*). *LTR7Y* was detected in 75% of the patients; however, it was less specific because it was observed in 24% of healthy controls (Fig. 6*F*). As observed for *ERVmap_1248*, circulating *LTR7Y* was independent of the age and sex (Supplementary Fig. 8D and E). The limitation of this investigation is that plasma samples did not match tumor samples.

Nevertheless, altogether, our data reveal that mesothelioma-specific ERV levels are associated with a better clinical outcome and can be detected in the blood.

## Discussion

In this study, we provide evidence that ERV expression is associated with clinical outcome and viral mimicry response in human mesothelioma, consistent with our observation in a mouse model of mesothelioma development in mice exposed to asbestos.^9^

This is the first time that a specific ERV is associated with clinical outcome in human PM. A previous study^17^ had identified the location of mesothelioma-specific expressed ERVs based on the comparison of TCGA data with the average expression of ERV in GTEX data set. Interestingly, the location of some of these ERV is near genes relevant for mesothelioma, such as *UPK3B* and *MSLN*. For example, *UPK3B* is a marker for mesothelial cells^39^ and high levels of *UPK3B* expression are associated with better OS.^5^^,^^6^ In addition, *UPK3B* and *MSLN* are significantly positively correlated with mesothelioma E-score.^8^ In our study, we used three different data sets and validated the enrichment of specific ERVs, including in the blood from the patients with PM.

Both selected solo-LTR and near full-length proviral sequences have elevated expression levels in mesothelioma. Three solo-LTR were most often enriched in the tumor tissues and mesothelioma primary cultures. Solo-LTR is known to act as an enhancer, and it is noteworthy that *LTR7Y* acts as stage-specific promoter in pluripotent epiblasts, one of three major cell types in the preimplantation blastocyst.^25^ *LTR7Y* expression is controlled by methylation^40^ and is frequently observed in enhancer regions in naive human embryonic stem cells,^41^ whereas it is not present in adult tissue.^25^ The observation that mesothelioma cells express pluripotent cell enhancers is consistent with our previous data where we had used a lentiviral fluorescence-based reporter construct sensing high SOX2 and OCT4 levels to identify and isolate a subpopulation of mesothelioma cells with cancer stem cell properties, characterized by chemoresistance and a higher tumor-initiating capacity in orthotopic xenograft and allograft mouse models.^42^ Future studies could take advantage of the new knowledge and use the recently described LTR7Y-driven reporter^43^ to further explore pluripotent mesothelioma cells.

Although not further investigated in this study, *LTR7Y* is in genomic regions enriched in retroposed genes, or genes linked to mesothelioma biology (e.g., S-score^8^), which is consistent with promoter or proximal enhancer effect of TE.^12^ Not much is known about the other two most often expressed solo-LTR, with the exception that a subset of *LTR48B* elements acquired enhancer activity in the pluripotent cells.^44^

Consistent with the enrichment in *LTR7Y*, the three ERVmap genes enriched in mesothelioma belong to the ERVH family, which is characterized by LTR7 promoter family and is expressed early in the embryo.^45^

In addition to embryogenesis, subsets of *LTR7* and *LTR7Y* elements are known to be up-regulated in oncogenic states due to promoter demethylation.^46^

A correlation of the *LTR7* transcriptional regulatory signals with human embryonic stem cell–specific expression of lncRNAs has been reported,^47^ including *linc-ROR* which is enriched in PM translatome.^26^ This is consistent with the observation that high level of transcription of several ERV loci promotes the expression of lncRNA,^48^ which seems important in controlling cell identity.^49^^,^^50^

Silencing of ERV in adult tissues occurs through binding of HERV-targeting KZFP, which recruit KAP1/TRIM28 co-repressor to induce heterochromatin formation.^41^ Therefore, the level of variation of HERVH-associated KZFP can potentially be the reason of differential expression of HERVH in PM. For example, the potential repressor ZNF534, which is particularly enriched in *LTR7* and which is associated with pluripotency,^45^ is up-regulated in sarcomatoid compared with epithelioid mesothelioma.^5^

The ERVK family was less enriched in mesothelioma. ERVKs are the only ERVs that are human specific with intact open-reading frames, reported to generate viral-like proteins in teratocarcinoma cell lines and human blastocysts.^51^ *ERVmap-k48* used as a control has a sequence of approximately 3900 bp encoding for *Gag* and is located near the housekeeping gene *SSBP1*, which has been hypothesized to drive its transcription and possibly explains the reason for stable levels in normal versus tumor tissue.^29^

KAP1/TRIM28 recruits chromatin modifiers including SETDB1, which is mutated in a subset of mesothelioma,^5^^,^^7^^,^^16^ thereby possibly also contributing to differential HERVH and HERVK expression.

According to the knowledge about epigenetic control of ERV expression,^52^ we observed that *ERVmap_1248* expression increases on inhibition of methylation in normal mesothelial cells. Induction of the expression of ERV has been documented in studies supporting the use of viral mimicry in clinical trials, where effects of immune checkpoint inhibitor are tested in combination with demethylating agents.^52^^,^^53^ Basal ERV expression was correlated with low methylation pattern in a pan-cancer analysis.^54^ Changes in DNA methylation have been documented in human mesothelioma (reviewed in Vandenhoeck et al.^55^), and we recently discussed^9^ other factors such as control of DNA methylation that is dysregulated in mesothelioma besides KZFP.

Consistent with the association between expression of transposable elements and the viral mimicry response observed in cancer in general,^28^ and our own observation in a mesothelioma development model,^9^ we observed a basal activation of type I IFN signaling in tumors expressing high levels of *ERVmap_1248*. Of note, based on the mRNA expression profile, mesothelioma tumors have been clustered into four groups.^5^^,^^7^ Pathway-enriched analysis of genes expressed in the clusters revealed, among others, enrichment of reactome antiviral mechanism by ISG in one of the TCGA clusters, and this is confirmed in the epithelioid group of Bueno et al.^5^ Patients with this profile have a better clinical outcome^7^^,^^9^^,^^15^ and *BAP1* mutations,^15^ consistent with our observations that tumors with high levels of *ERVmap_1248* are associated with *BAP1* mutations.

Mesothelioma cells were previously found to maintain the activation of the type I IFN signaling pathway.^9^^,^^56^ In addition, mesothelioma was described as being a cancer highly enriched for the 38-ISG signature, not always justified by the presence of immune cells in the microenvironment.^57^ We put forward the hypothesis that ERV expression reactivation in selected samples is a possible cause for type I IFN activation. These tumors are most likely associated with *BAP1* but not *CDKN2A/B* mutations if we consider that *IFNB1* and all the 15 other type I IFN genes are co-deleted in a large fraction of tumors bearing *CDKN2A/B* deletions.^34^

Activation of type I IFN was associated with response to immune checkpoint blockade in clear cell renal cancer.^58^ ERV expression is a predictor of patient response to immunotherapy in a urothelial cancer cohort,^30^ and, interestingly, in that study, it was a better predictor compared with type I IFN signature. Investigation of the expression of 66^59^ ERVs revealed that some ERVs are associated with both immune activation and checkpoint pathway up-regulation in clear cell renal cell carcinoma^60^ and expression levels of one of those ERVs predicted response to checkpoint blockade. In addition, high ERV expression was associated with better overall clinical outcome in a cohort of patients with melanoma whereas repression of ERV was observed in the cohort with worst outcome.^61^ Furthermore, *ERVmap_2637* expression was higher in patients with melanoma with complete response to anti–programmed cell death protein 1 treatment^62^ and negatively correlated with *KDM5B* expression, which recruits SETDB1. Therefore, our observations are also important for mesothelioma therapy. Indeed, therapeutic approaches exploiting type I IFN pathway signaling have already been implemented in the clinic^63^ or proposed on the basis of preclinical studies.^64^^,^^65^ Future studies may investigate whether ERV expression could be a predictor of sensitivity to those therapeutic approaches and immune checkpoint inhibition, although it should be taken into account for therapies inducing type I IFN signaling that some mesothelioma have lost type I IFN genes^34^ and might therefore not be able to activate such signaling. ERV expression could be, for example, helpful to stratify patients with epithelioid histotype, which overall respond less well to immune checkpoint inhibition than those with sarcomatoid histotype.^66^

## CRediT Authorship Contribution Statement

**Suna Sun:** Validation, Formal analysis, Investigation, Data curation, Writing—original draft, Writing—review and editing, and Visualization.

**Weihong Qi:** Methodology, Software, Validation, Formal analysis, Data curation, and Writing—review and editing.

**Hubert Rehrauer:** Conceptualization, Methodology, Software, Validation, and Writing—review and editing.

**Manuel Ronner:** Investigation and Writing—review and editing.

**Ananya Hariharan:** Investigation and Writing—review and editing.

**Martin Wipplinger:** Investigation and Writing—review and editing.

**Clément Meiller:** Formal analysis and Writing—review and editing.

**Rolf Stahel:** Resources and Writing—review and editing.

**Martin Früh:** Resources and Writing—review and editing.

**Ferdinando Cerciello:** Resources and Writing—review and editing.

**Jean-François Fonteneau:** Conceptualization, Methodology, and Writing—review and editing.

**Didier Jean:** Methodology and Writing—review and editing.

**Emanuela Felley-Bosco:** Conceptualization, Methodology, Formal analysis, Writing—original draft, Writing—review and editing, Supervision, and Funding acquisition.
