# Supplementary material for: Viral Mimicry Response Is Associated With Clinical Outcome in Pleural Mesothelioma
Source: JTO Clin Res Rep. 2022 Nov 7;3(12):100430. doi: 10.1016/j.jtocrr.2022.100430 (PMC9709230; doi:10.1016/j.jtocrr.2022.100430)
Supplement: Supplementary Methods [file mmc1.docx]

**Viral mimicry response is associated with clinical outcome in pleural mesothelioma**

Suna Sun^1^, Weihong Qi^2^, Hubert Rehrauer^2^, Manuel Ronner^1^, Ananya Hariharan^1^, Martin Wipplinger^1^, Clément Meiller^3^, Rolf Stahel^4,5^, Martin Früh^5,6,7^, Ferdinando Cerciello^6^, Jean-François Fonteneau^8^, Didier Jean^3^, Emanuela Felley-Bosco^1^

**Supplementary Figures**


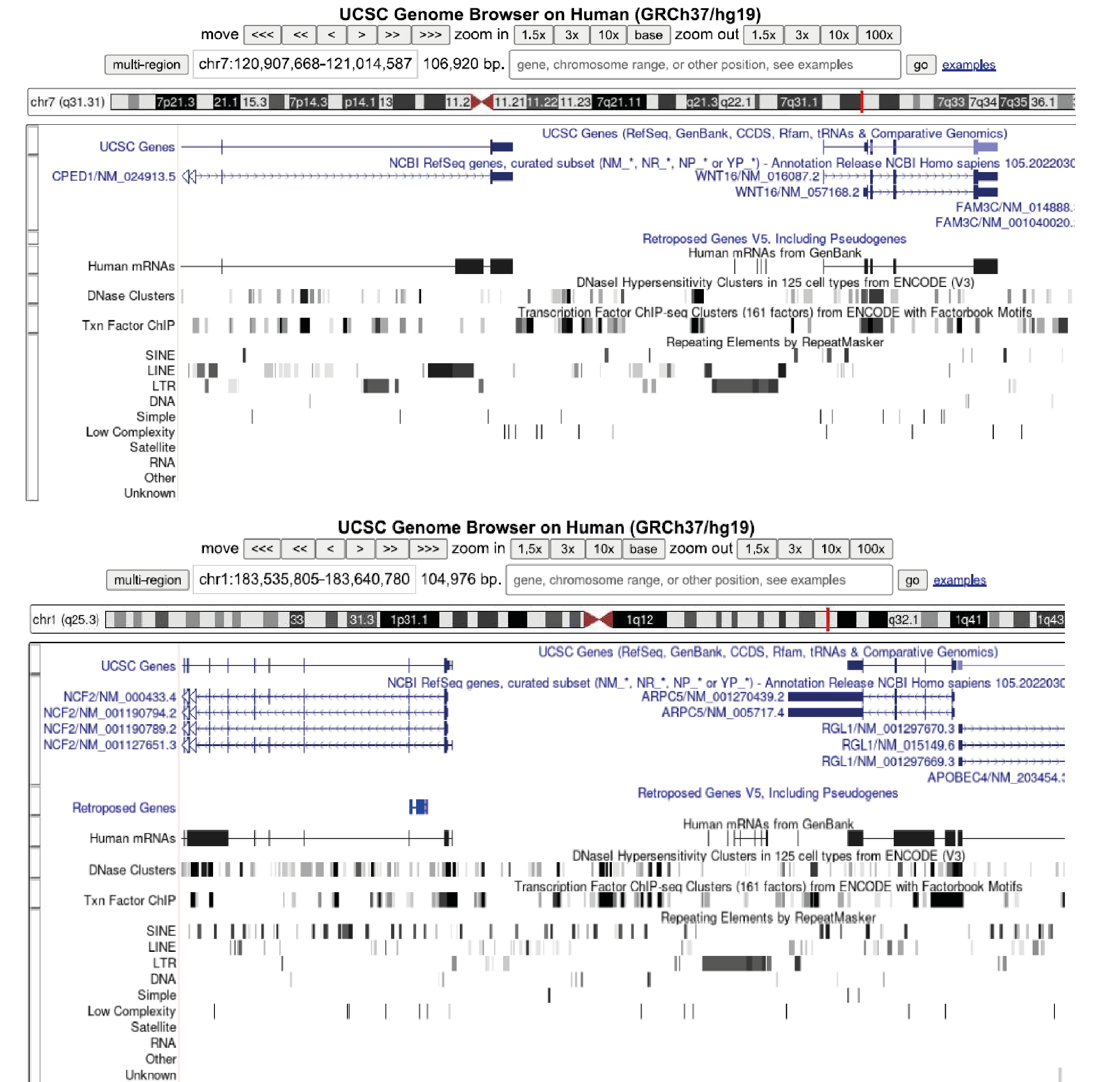


**Figure S1. LTR7Y upregulated in blastocyst stage are located near genes relevant to PM.** Screenshots of the UCSC Genome Browser show two different LTR7Y upregulated in blastocyst stage near genes such as WNT16 and FAM3C , which are enriched in PM translatome (1) or NCF2 which is correlated to the so-called S-score, which defines the sarcomatoid component proportion of PM by transcriptome analysis (2)**.**

**
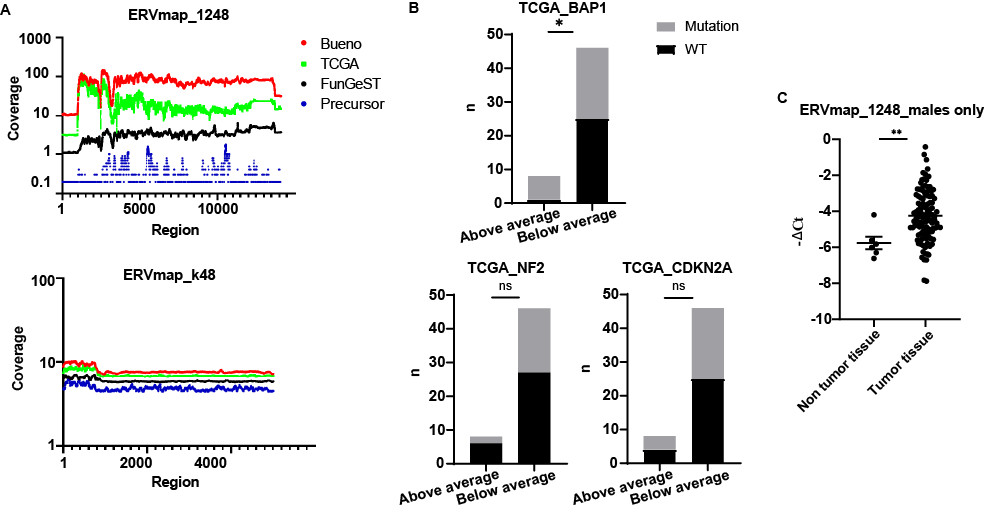
**

**Figure S2. *ERVmap_1248* is enriched in PM with BAP1 genetic alterations.** (A) *ERVmap_1248* and *ERVmap_k48* counts coverage in tumor tissue (Bueno and TCGA datasets), primary PM cultures (FunGeST) and mesothelial precursors. (B) Tumors with *ERVmap_1248* levels above the average are enriched in samples with BAP1mutations, while no significant enrichment is observed for *NF2* or *CDKN2A* mutations. (*) P<0.05, Chi-square test. C) *ERV_map1248* expression is higher in tumor compared to non-tumor tissues in males.


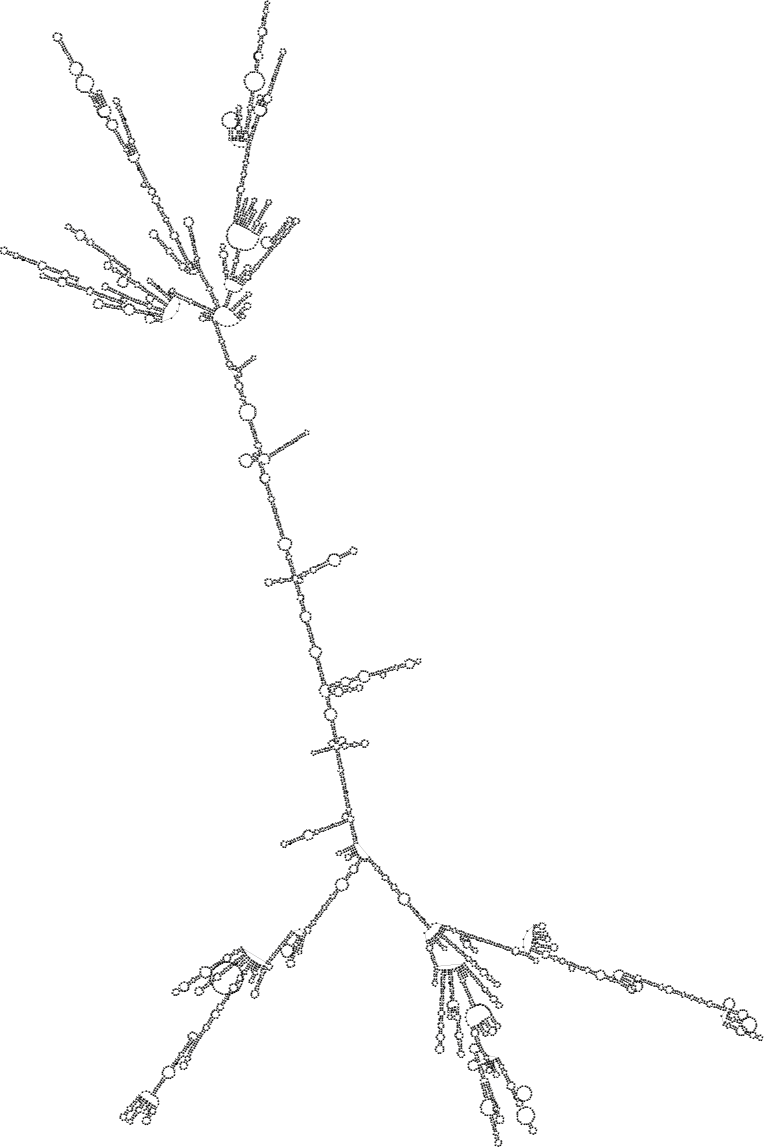


**Figure S3.** **Secondary structure prediction of *ERVmap_1248***. The structure was created by RNAfold web server:http://rna.tbi.univie.ac.at/cgi-bin/RNAWebSuite/RNAfold.cgi


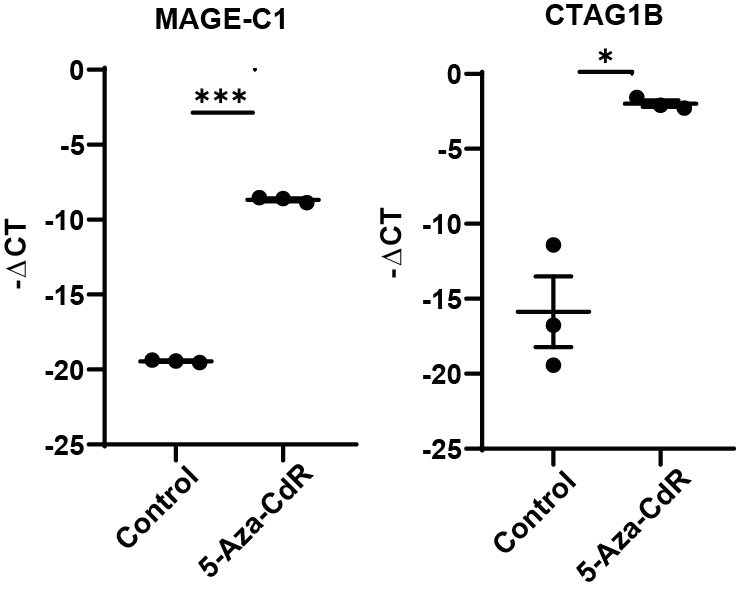


**Figure S4. Treatment of SDM103T2 cells with 5-Aza-CdR results in strong upregulation of *MAGE-C1* and *CTAG1B* expression**. These two genes are used as positive control (3). (*) P<0.05, (***) P<0.001, paired t test.


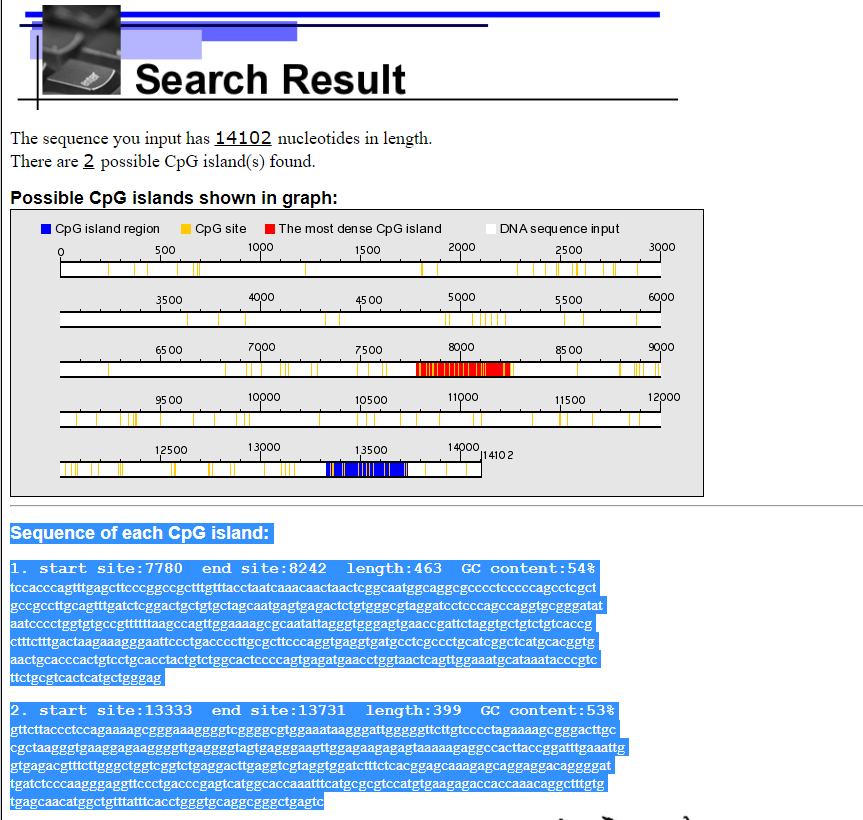


**Figure S5.** CpG islands in *ERVmap_1248* LTR. Analysis of in *ERVmap_1248* LTR (<http://dbcat.cgm.ntu.edu.tw/>) indicate the presence of two CpG islands. The most dense CpG island was selected for methylation analysis.


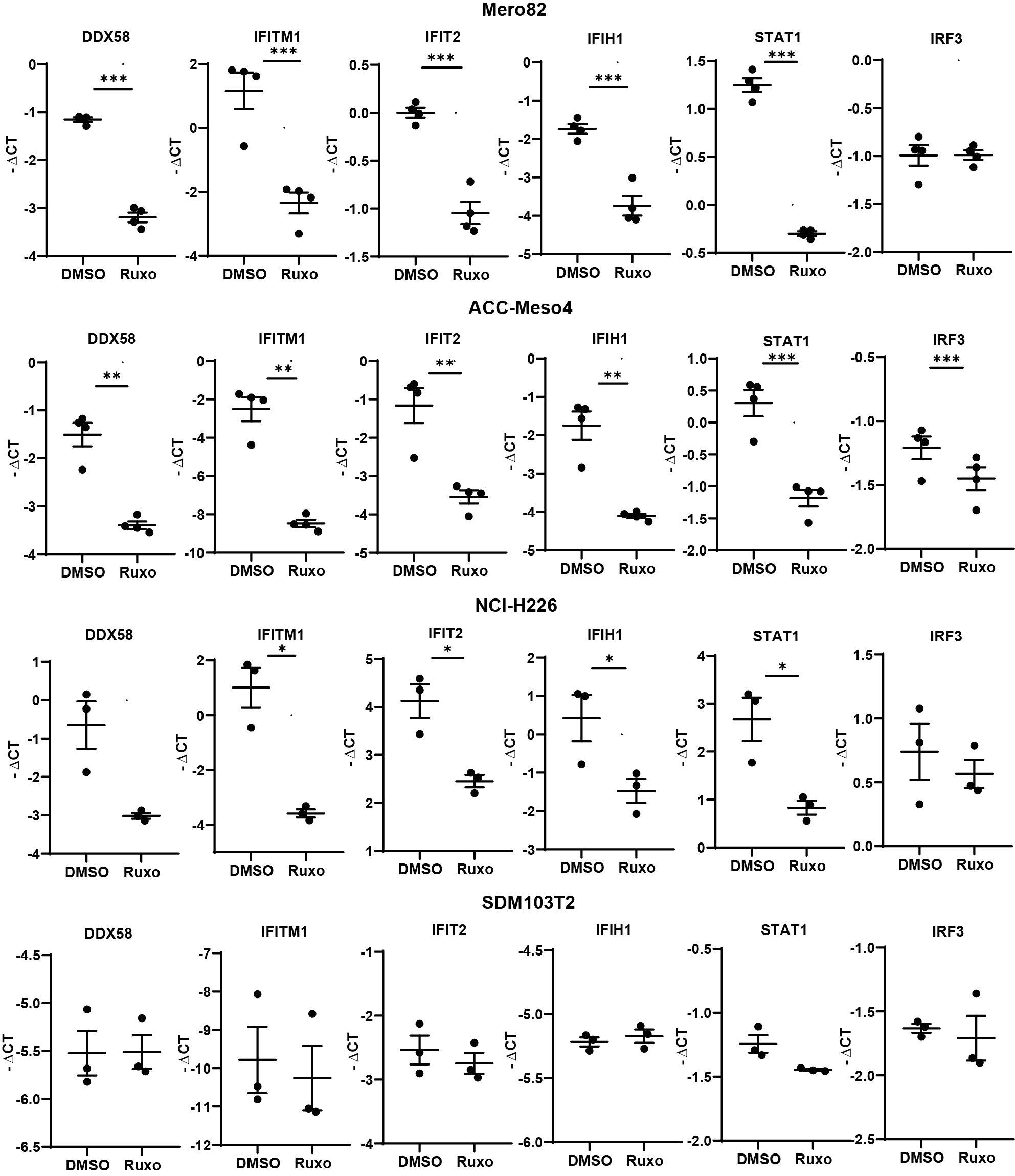


**Figure S6. Ruxolitinib (Ruxo), a JAK1/2 inhibitor, inhibits the expression of ISG in human mesothelioma cells.** The expression of ISG decreased after Ruxo treatment in PM cells bearing *IFNB1* wild-type but not in SDM103T2 where *IFNB1* is mutated. (*) P<0.05, (**) P<0.01, (***) P<0.001, paired t test.


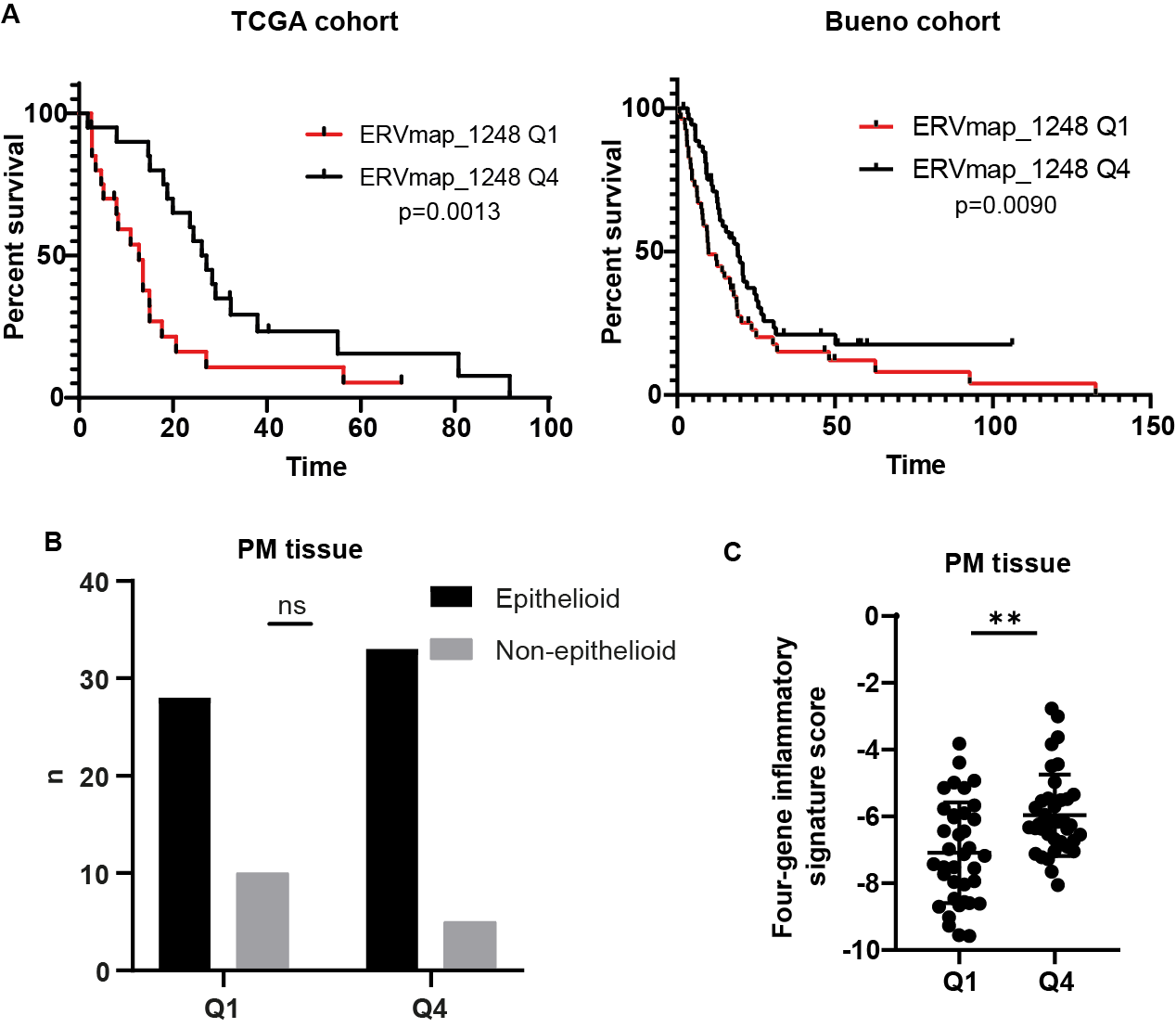


**Figure S7. High *ERVmap_1248* expression is associated with best OS in both TCGA and Bueno datasets**. (A) Kaplan-Meier curves of overall survival according to *ERVmap_1248* expression in PM patients from TCGA and Bueno cohorts. Red and black curves represent lower (Q1, TCGA: n=20, Bueno: n=53) or higher ERVmap_1248 (Q4, TCGA: n=20, Bueno: n=53) expression respectively. PM patients from Q4 have better survival rate than Q1 in both cohorts (TCGA: Q4 versus Q1: median OS 26.65 versus 12.72 months, HR = 2.746, p = 0.0013; Bueno: Q4 versus Q1: median OS 19.17 versus 9.863 months, HR = 1.584, p = 0.0090). Gehan-Breslow-Wilcoxon test. (B) Histo-type distribution has no difference between Q1 and Q4 in PM tissues. Ns= Not significant, Fisher’s exact test. (C) Four-gene inflammatory signature(4) score is significantly higher in *ERVmap_1248* high PM tissues (Q4). (**) P<0.01, Mann-Whitney test.


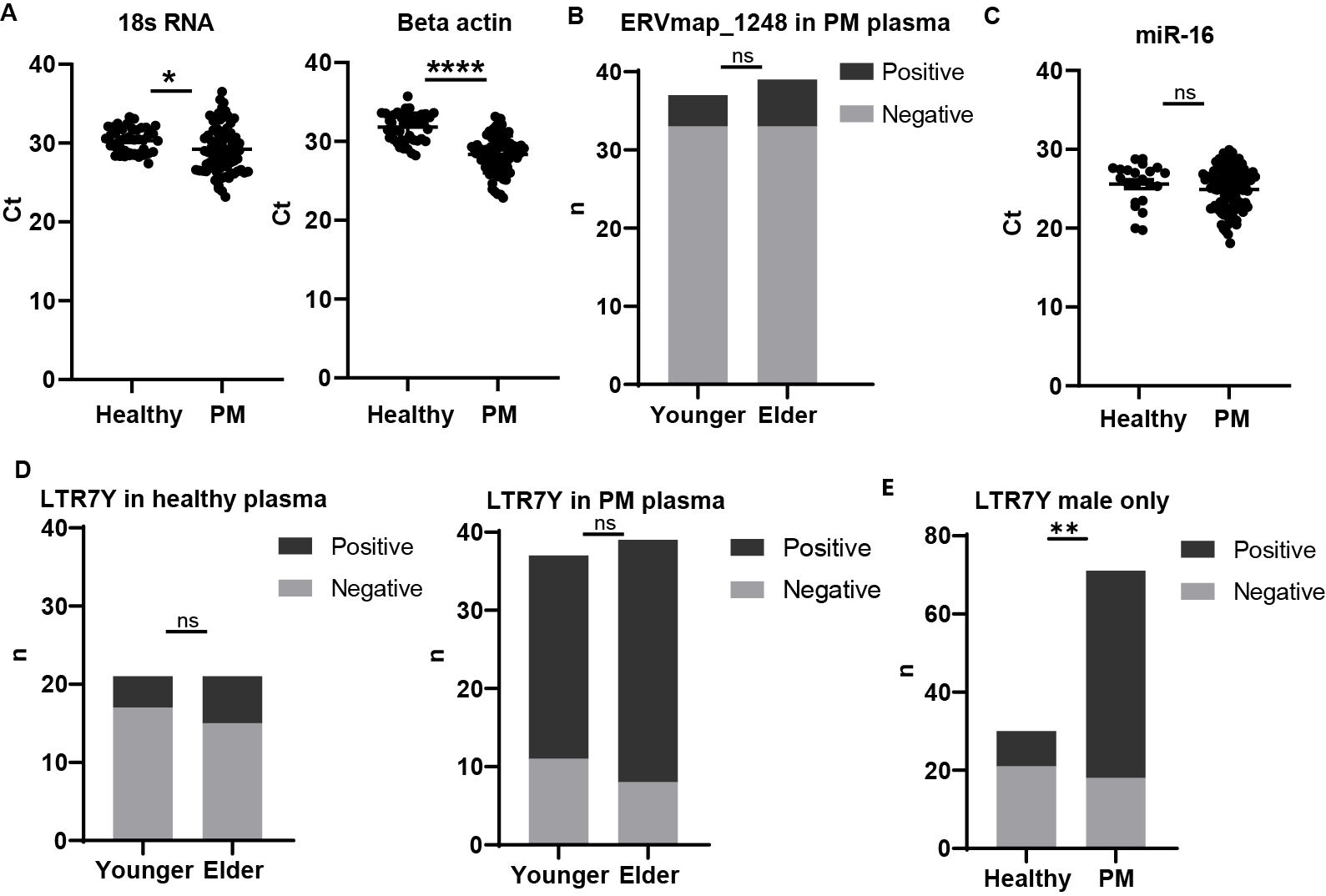


**Figure S8. Reference genes expression levels in plasma and circulating *ERVmap_1248* and *LTR7Y* are independent of the age.** (A) Ct (cycle threshold) values of two reference genes 18S RNA and β-actin in 42 healthy and 76 PM patients plasma samples measured by q-PCR. (*) P<0.05, (****) P<0.0001, unpaired t test. (B) *ERVmap_1248* detection in the plasma of PM patients, groups were divided by median age. Fisher’s exact test. (C) Ct values of miR-16 in plasma samples used as miR-625-3p normalizer. Un-paired t test. (D) *LTR7Y* detection in the plasma of healthy donors and PM patients, groups were divided by median age. Fisher’s exact test. (E) *LTR7Y* detection in the plasma of male healthy donors and PM patients, females were all excluded. (**) P<0.01. Fisher’s exact test.

**References**

1. Grosso S, Marini A, Gyuraszova K, Voorde JV, Sfakianos A, Garland GD, et al. The pathogenesis of mesothelioma is driven by a dysregulated translatome. Nat Commun. 2021;12(1):4920.

2. Blum Y, Meiller C, Quetel L, Elarouci N, Ayadi M, Tashtanbaeva D, et al. Dissecting heterogeneity in malignant pleural mesothelioma through histo-molecular gradients for clinical applications. Nat Commun. 2019;10(1):1333.

3. Kresoja-Rakic J, Kapaklikaya E, Ziltener G, Dalcher D, Santoro R, Christensen BC, et al. Identification of cis- and trans-acting elements regulating calretinin expression in mesothelioma cells. Oncotarget. 2016;7(16):21272-86.

4. Peters S, Scherpereel A, Cornelissen R, Oulkhouir Y, Greillier L, Kaplan MA, et al. First-line nivolumab plus ipilimumab versus chemotherapy in patients with unresectable malignant pleural mesothelioma: 3-year outcomes from CheckMate 743. Ann Oncol. 2022;33(5):488-99.
