# Supplementary material for: Viral Mimicry Response Is Associated With Clinical Outcome in Pleural Mesothelioma
Source: JTO Clin Res Rep. 2022 Nov 7;3(12):100430. doi: 10.1016/j.jtocrr.2022.100430 (PMC9709230; doi:10.1016/j.jtocrr.2022.100430)
Supplement: Supplementary Methods [file mmc5.docx]

**Viral mimicry response is associated with clinical outcome in pleural mesothelioma**

Suna Sun^1^, Weihong Qi^2^, Hubert Rehrauer^2^, Manuel Ronner^1^, Ananya Hariharan^1^, Martin Wipplinger^1^, Clément Meiller^3^, Rolf Stahel^4,5^, Martin Früh^5,6,7^, Ferdinando Cerciello^6^, Jean-François Fonteneau^8^, Didier Jean^3^, Emanuela Felley-Bosco^1^

**Supplementary material and methods**

*RNA extraction, cDNA synthesis and RT-qPCR*

0.5 µg of total RNA was extracted from cells or tissues using RNeasy isolation kit (QIAGEN, Cat No.74106) and reverse-transcribed using the Quantitect Reverse Transcription Kit (QIAGEN, Cat No.205311) according to the manufacturer’s instructions.

Synthesized cDNA was diluted 1:60 and used for real-time quantitative PCR (RT-qPCR). SYBR green (Thermo Fisher, Cat No.4367659) and gene specific primers (sequences in Supplementary Table 2) were used for PCR amplification and detection on a 7500 FAST Real-Time PCR System (Applied Biosystems, Thermo Fisher Scientific) or QuantStudio 5 Real-Time PCR System. Relative mRNA levels were determined by comparing the PCR cycle thresholds between cDNA of a specific gene and histones (ΔCt)^1^.

Four-gene inflammatory signature score^2^ was calculated based of the average of the their ΔCt.

For detection of *ERVmap_1248*, *LTR7Y^3^*, *18S* *RNA* and *beta actin* in plasma, 5 μL of total RNA extracted using Qiazol and RNeasy kit from 200 μL of plasma was reverse transcribed. Synthesized cDNA was diluted 1:30 and used for qPCR as mentioned above using gene specific primers (sequences in Supplementary Table 2).

For detection of miRNAs, the reverse transcription was performed using miScript II RT Kit (Qiagen) using 5 μL of total RNA, the details about reverse transcription and qPCR was previously described ^4^. Since hemolysis can happen during plasma preparation and processing we measured plasma levels of miR-451-3p and miR-23a as hemolysis indicators ^5^. Samples, which had ΔCt (miR-23a – miR-451-3p) more than 5 Ct cycles were excluded as samples contamination induced by hemolysis.

*Cell culture and drug treatment*

Mesothelioma cell lines NCI-H226 (RRID:CVCL_1544), Mero82 (RRID:CVCL_2594), ACC-Meso4 (RRID:CVCL_5114), SDM103T2(CVCL_D313) and normal mesothelial cells LP9/TERT-1 (RRID:CVCL_E108) and SDM104 (RRID:CVCL_IT34) ^6^ were cultured as previously described ^7^ at 37°C in a humidified 5% CO_2_ incubator. Cell lines were confirmed to be free of Mycoplasma on a regular basis using PCR Mycoplasma kit (MD Bioproducts). Cells were seeded in 6-well plates and after 24 hours were treated with 250nM 5-Aza-2’-deoxycytidine (5-Aza-CdR) (Selleckchem, Cat No.S1200) or 1μM Ruxolitinib (Selleckchem, Cat No.S1378) or DMSO as mock, the procedure of treatment and collection was previously described ^8^.

*DsRNA pull-down*

8 μg of purified RNA was added to 200 μl fresh prepared immunoprecipitation (IP) buffer (50 mM Tris-HCl pH 8, 150 mM NaCl, 1% Triton X-100, and 1 mM EDTA), supplemented with 20 U/ml RNase inhibitor. Pre-clearing of the unspecific binding was done by incubating the 8 μg RNA in IP buffer with 10 μl Protein A Sepharose (Bio Vision Cat.No.6501-5) for 30 min at 4°C on a rotating wheel. After centrifugation (14,000 g at 4°C for 5 min), 95 ng RNA was taken from the supernatant to determine input. The remaining supernatant was then equally divided into two parts, one for the anti-dsRNA antibody J2 ^9^ (SCICONS,clone J2, No.10010200, RRID:AB_2651015) and the other for the IgG control of the same isotype. 1 μg of J2 or IgG antibody was added into the supernatant, followed by incubation overnight on a rotating wheel at 4°C. 5μl Protein A Sepharose was then added into each reaction, followed by incubation for another 4 hours at 4°C. Next, the beads were collected and washed with 500 μl pre-chilled washing buffer (50 mM Tris-Cl, pH 7.5, 150 mM NaCl) for 3 times. Finally, the co-precipitated RNA on the beads were purified using Qiazol and miRNeasy kit as above.

*Protein extraction and Western Blotting*

Total protein extracts were obtained by lysing the cells with hot Laemmli buffer (60 mM
Tris-HCl pH 6.8, 100 mM DTT, 5% glycerol, 1.7% SDS) and passed through syringes (26G) ^10^. Protein concentration was determined using a Pierce™660nm Protein Assay (Thermo Fisher Scientific). A total of 5 μg protein extract was separated on denaturing 15% SDS-PAGE gels and proteins were transferred onto PVDF membranes (0.45 μm, Perkin Elmer, Waltham, MA). Membranes were probed with the following primary antibodies: MDA5 (Cell Signaling Technology Cat# 5321, RRID: AB_10694490), RIG-I (Cell Signaling Technology Cat# 3743, RRID:AB_2269233), STAT1 (Cell Signaling Technology Cat# 14994, RRID: AB_2737027), IRF3 (Cell Signaling Technology Cat# 4302, RRID: AB_1904036), MAVS (Cell Signaling Technology Cat# 3993, RRID: AB_823565), ISG15 (Santa Cruz Biotechnology Cat# sc-166755, RRID:AB_2126308) and β-actin (C4, MP Bio-medicals MP691002 RRID:AB_2335127), Membranes were then incubated with one of the following secondary antibodies: rabbit anti-mouse IgG-HRP (no. A9004) or goat anti-rabbit IgG-HRP (no. A0545), obtained from Sigma Aldrich. The signals were detected by enhanced chemiluminescence (Clarity TM ECL Substrate, BioRad, Hercules, CA) using Fusion Digital Imager (Vilber Lourmat, Marne-la-Vallée, France). Quantification was done using ImageJ software.

*Genomic DNA extraction from cells and human tissues*

Genomic DNA (gDNA) was extracted from 0.5-1 Mio cells using the DNeasy Blood&tissue kit (QIAGEN, CAT No.69504) according to manufacturer’s instructions.

gDNA extraction from human non-tumor or tumor tissues was performed as follows: around 20mg frozen tissue was resuspended in 180 μl ATL Buffer followed by addition of 20 μl proteinase K and incubation at 56 °C, 450 rpm, until the tissue was completely lysed (around 1 hour). Then 180 μl ATL Buffer + 20 μl proteinase K were added again followed by another 5-10 minutes incubation at 56 °C. After vigorous vortexing to homogenize the mixture, 800 μg RNase A were added and incubated for 2 minutes at room temperature. Afterwards, gDNA was extracted using the “DNeasy Blood & Tissue” kit (QIAGEN, Cat No.69504) according to manufacturer’s instructions.

*Genomic PCR*

PCR to amplify the region of *IFNB1* was performed as previously described ^11^ in a total volume of 25 μl containing 1x GoTaq G2 Hot Start Green Master Mix (Promega), 0.5 mM of each primer, and 10 ng of gDNA from cells. PCR program was used with one cycle during 2 minutes at 95 C, then 35 cycles of amplification, 1minute at 94 C, 45 seconds at 50 C and 45 seconds at 72 C, after amplification a final extension of 5 minutes at 72 C. Products were confirmed by electrophoresis on a 2% agarose gel and visualized under UV 365nM (VILBER LOURMAT, serial No.13200087).

PCR to amplify the region of exon 5 of *BAP1* was performed as previously described ^12^ in a total volume of 25 μl containing 1x cloned PFU Reaction Buffer (Stratagene), 6% DMSO, 0.25 mM PCR Nucleotide Mix, 0.5 mM of each primer, 1.25 U of Cloned PFU DNA polymerase (Stratagene) and 10 ng of gDNA from cells. Products were confirmed by electrophoresis on a 4% agarose gel and visualized under UV 365nM. The primers used are listed in supplementary Table 2.

*RNA interference*

In order to down-regulate mitochondrial antiviral signaling protein (MAVS) expression, ON-TARGETplus SMARTpool or single siRNAs against *MAVS* or siGENOME Non-Targeting siRNA pool #2 and DharmaFECT 1 transfection reagent were obtained from Dharmacon. siRNA dissolved in 1X siRNA buffer (Dharmacon) was combined with transfection reagent dissolved in OptiMEM (final concentration 0.084%) and incubated for 20 min. Then, cells resuspended in normal growth medium were added to the siRNA/DharmaFECT 1 mixture and seeded onto plates, allowing for a final siRNA concentration of 10 nM. 0.4 × 10^5^ cells (12-well plate) were plated for whole cell protein lysates as wells as RNA extraction 96 hours later.

*Bisulfite treatment for gDNA and qMSP*

To perform methylation studies, 200-500ng gDNA from cells or frozen tissue was subjected to sodium bisulfite treatment performed using the EZ DNA Methylation Gold™ Kit (Zymo Research,Cat No. D5005 & D5006) according to manufacturer’s instructions, with an extra step of incubating the samples for 7 minutes at 95C before adding CT Conversion Reagent solution. Measurement was performed by quantitative methylation specific PCR (qMSP). Methylated (“M”) - and unmethylated (“U”)-specific primers (supplementary Table 2) were designed within CpG islands surrounding the promoter of *ERVmap_1248* qPCR amplicon region by using the online platform MethPrimer ^13^ as per standard qMSP design guidelines. Commercial universal methylated (ZYMO RESEARCH, Cat.#D5011-1) and universal unmethylated human DNA (ZYMO RESEARCH, Cat.#D5014-1) were used to generate absolute methylation and unmethylation standard curves by performing bisulfite conversion and qMSP with designed *ERVmap_1248* “M” and “U” primers. The copy numbers of methylated and unmethylated sequences for *ERVmap_1248* promoter were both established by extrapolation from the standard curves. The percentage of methylation was defined as the ratio between methylated molecules and the sum of methylated and unmethylated molecules ^14^. End point qMSP products were run on a 4% agarose gel and visualized under UV 365nM and excised. After purification according to the Macherey-Nagel NucleoSpin® Gel and PCR Clean-up protocol, products were sent for Sanger sequencing (done by Microsynth AG, Balgach, Switzerland). Sequencing confirmed that the “M” primers recognized a fully methylated product, while the “U” primers recognized a fully demethylated product in gDNA from cells and tissue.

*Blood collection, preparation of plasma and RNA Extraction*

Venous blood samples from PM patients were collected before chemotherapy treatment. Within 1 hour of

collection, the blood was centrifuged in the original vials for 10 min at 3000 rpm (approx. 1600 x g)at room temperature. Plasma was carefully transferred to a new 15 mL non-pyrogenic Falcon tubeand centrifuged again for 10 min at 3000 rpm at room temperature in order to pellet any residual cells.Cell-free plasma was then aliquoted to 0.5 mL volumes in 1.5 mL RNase-free Eppendorf tubes andstored at -80 C for further processing. Total RNA enriched with circulating miRNA was extracted from 200μL plasma using the miRNeasy Mini kit (Qiagen, Hilden, Germany) following the manufacturer’s instruction. Plasma samples were thawed completely on ice and mixed gently by pipetting immediately prior to aliquoting for RNA isolation and to evenly disperse any particulates present. 5 volumes of QIAzol Lysis Reagent were added followed by vortexing for dissociation of nucleoprotein complexes and 5 min incubation at room temperature. One ug MS2 carrier RNA (Roche, Basel, Switzerland) was added before addition of 1 volume of chloroform, followed by vigorously vortexing and centrifugation for 15 min at 12,000g at 4C for subsequent phase separation. The upper aqueous phase containing total RNA was carefully transferred to a new collection tube, and 1.5 volume of 100% ethanol was added. The sample was passed through the RNeasy Mini spin column and purified by several washing steps. The RNA was eluted from the spin column using two subsequent RNase-free water aliquots (15 μL each), and stored at -80C for further processing.

**References**

1. Andre M, Felley-Bosco E. Heme oxygenase-1 induction by endogenous nitric oxide: influence of intracellular glutathione. *FEBS Lett* 2003;546:223-227.

2. Peters S, Scherpereel A, Cornelissen R, et al. First-line nivolumab plus ipilimumab versus chemotherapy in patients with unresectable malignant pleural mesothelioma: 3-year outcomes from CheckMate 743. *Ann Oncol* 2022;33:488-499.

3. Fu Y, Zhou Z, Wang H, et al. IFITM1 suppresses expression of human endogenous retroviruses in human embryonic stem cells. *FEBS Open Bio* 2017;7:1102-1110.

4. Kresoja-Rakic J, Szpechcinski A, Kirschner MB, et al. miR-625-3p and lncRNA GAS5 in Liquid Biopsies for Predicting the Outcome of Malignant Pleural Mesothelioma Patients Treated with Neo-Adjuvant Chemotherapy and Surgery. *Noncoding RNA* 2019;5.

5. Blondal T, Jensby Nielsen S, Baker A, et al. Assessing sample and miRNA profile quality in serum and plasma or other biofluids. *Methods* 2013;59:S1-6.

6. Echeverry N, Ziltener G, Barbone D, et al. Inhibition of autophagy sensitizes malignant pleural mesothelioma cells to dual PI3K/mTOR inhibitors. *Cell Death Dis* 2015;6:e1757.

7. Okonska A, Buhler S, Rao V, et al. Functional Genomic Screen in Mesothelioma Reveals that Loss of Function of BRCA1-Associated Protein 1 Induces Chemoresistance to Ribonucleotide Reductase Inhibition. *Mol Cancer Ther* 2020;19:552-563.

8. Sun S, Frontini F, Qi W, et al. Endogenous retrovirus expression activates type-I interferon signaling in an experimental mouse model of mesothelioma development. *Cancer Lett* 2021;507:26-38.

9. Weber F, Wagner V, Rasmussen SB, et al. Double-stranded RNA is produced by positive-strand RNA viruses and DNA viruses but not in detectable amounts by negative-strand RNA viruses. *J Virol* 2006;80:5059-5064.

10. Kresoja-Rakic J, Kapaklikaya E, Ziltener G, et al. Identification of cis- and trans-acting elements regulating calretinin expression in mesothelioma cells. *Oncotarget* 2016;7:21272-21286.

11. Delaunay T, Achard C, Boisgerault N, et al. Frequent Homozygous Deletions of Type I Interferon Genes in Pleural Mesothelioma Confer Sensitivity to Oncolytic Measles Virus. *J Thorac Oncol* 2020;15:827-842.

12. Rusch A, Ziltener G, Nackaerts K, et al. Prevalence of BRCA-1 associated protein 1 germline mutation in sporadic malignant pleural mesothelioma cases. *Lung Cancer* 2015;87:77-79.

13. Li LC, Dahiya R. MethPrimer: designing primers for methylation PCRs. *Bioinformatics* 2002;18:1427-1431.

14. Furlan C, Polesel J, Barzan L, et al. Prognostic significance of LINE-1 hypomethylation in oropharyngeal squamous cell carcinoma. *Clin Epigenetics* 2017;9:58.
